# The Effect of Body Mass Index on Peak Growth Hormone Response During Stimulation Testing: A Retrospective Analysis

**DOI:** 10.3390/children13101345

**Published:** 2026-10-03

**Authors:** Eleonora Rulli, Marcello Candelli, Donato Rigante, Clelia Cipolla, Ignazio Cammisa

**Affiliations:** 1 Department of Life Sciences and Public Health, Fondazione Policlinico Universitario A. Gemelli IRCCS, 00168 Rome, Italy; eleonora.rulli01@icatt.it (E.R.); donato.rigante@unicatt.it (D.R.); clelia.cipolla@policlinicogemelli.it (C.C.); 2 Department of Emergency Anesthesiological and Reanimation Sciences, Fondazione Policlinico Universitario A. Gemelli IRCCS, 00168 Rome, Italy; marcello.candelli@policlinicogemelli.it; 3 Department of Pediatrics, Università Cattolica Sacro Cuore, 00168 Rome, Italy; 4 Department of Paediatrics, San Giovanni Evangelista Hospital, Tivoli, 00019 Rome, Italy

**Keywords:** growth hormone deficiency, children, diagnosis, body mass index, stimulation testing

## Abstract

**Highlights:**

**What are the main findings?**

- Higher BMI SDS was independently associated with lower peak GH levels during stimulation testing in children evaluated for suspected GHD.
- Each 1-SDS increase in BMI was associated with a 1.63 ng/mL reduction in peak GH and 2.10-fold higher odds of confirmed GHD.

**What are the implications of the main findings?**

- The inverse association between BMI SDS and peak GH was significant in children with confirmed GHD but not in those without GHD.
- BMI and adiposity should be considered when interpreting GH stimulation tests, particularly in children with borderline GH responses.

**Abstract:**

**Background**: Growth hormone (GH) stimulation testing remains the diagnostic gold standard for GH deficiency (GHD), but body mass index (BMI) inversely influences peak GH levels, creating a risk of overdiagnosis. We evaluated the association between BMI standard deviation score (SDS) and peak GH response in children undergoing diagnostic work-up for suspected GHD. **Methods**: This is a retrospective single-center study of 298 children (55.4% male, mean age 9.5 ± 2.9 years) evaluated for growth failure with arginine or clonidine stimulation testing. GHD was confirmed in 114 patients (38.3%). Multivariable linear and logistic regression models evaluated associations after adjustment for age, sex, and pubertal status. **Results**: BMI SDS negatively correlated with peak GH levels in the overall cohort (r = −0.299, *p* < 0.001) and in GHD patients (r = −0.322, *p* < 0.001), but not in non-GHD controls (r = −0.102, *p* = 0.167; Fisher’s z *p* = 0.050). On multivariable analysis, each 1-SDS increase in BMI SDS was independently associated with a 1.63 ng/mL reduction in peak GH (β = −1.63, 95% CI −2.20 to −1.07; *p* < 0.001) and 2.10-fold higher odds of GHD diagnosis (OR 2.10, 95% CI 1.60–2.74; *p* < 0.001). Across BMI SDS quartiles (Q1 to Q4), mean peak GH progressively decreased from 11.14 to 6.93 ng/mL (*p* < 0.0001), while GHD prevalence increased from 22.4% to 62.7% (*p* < 0.0001). **Conclusions**: BMI SDS significantly blunts peak GH response and increases the likelihood of a GHD diagnosis. This inverse relationship was evident primarily in children with confirmed GHD, suggesting that adiposity predominantly impairs an already compromised somatotropic axis. Adiposity should be accounted for when interpreting GH stimulation tests.

## 1. Introduction

Growth hormone deficiency (GHD) is one of the leading causes of impaired linear growth and short stature in children [1,2,3]: it is characterized by insufficient secretion of growth hormone (GH) from the anterior pituitary gland, leading to reduced production of insulin-like growth factor 1 (IGF-1) and consequently leading to delayed skeletal maturation, increased adiposity, and reduced muscle mass [2,3,4]. Although the classic phenotype of GHD includes short stature (height ≤−2 standard deviation scores [SDS]), frontal bossing, midfacial hypoplasia, and truncal adiposity, some patients may present solely with reduced growth velocity or isolated short stature [3,4,5]. Consequently, distinguishing GHD from other causes of impaired growth can be challenging [5]. Although the diagnosis of GHD is supported by auxological assessment and the measurement of circulating IGF-1 and insulin-like growth factor-binding protein 3 (IGFBP-3) levels, the biochemical evaluation of GH secretion using pharmacological stimulation tests remains the diagnostic gold standard [6,7,8]. The final diagnosis of GHD requires a suboptimal response in two separate GH stimulation tests [8,9,10]. However, these tests have several well-recognized limitations, including poor reproducibility, inter-assay variability, lack of universally accepted GH cut-off values, and susceptibility to multiple physiological and clinical confounding factors as age, puberty, nutritional status, and body weight [6,7,8,9,10,11].

In recent years, increasing attention has been devoted to the role of body mass index (BMI) on the results of GH stimulation tests. Several studies have consistently demonstrated an inverse relationship between BMI and peak GH levels, suggesting that adiposity could significantly affect the interpretation of such tests [12]. Even if the underlying mechanisms have not been fully explained, several pathophysiological processes have been proposed [12,13,14]. Increased adiposity is associated with a reduction in the frequency and amplitude of GH secretory bursts, as well as enhanced GH clearance [12,13,14]. Hyperinsulinemia may directly inhibit pituitary GH synthesis and secretion or indirectly increase circulating IGF-1 levels by reducing hepatic production of IGF-binding protein 1. Elevated free fatty acid concentrations may further suppress GH release, at least in part through increased somatostatinergic tone [12,13,14,15,16,17]. In addition, higher circulating levels of growth hormone-binding protein (GHBP), which are positively associated with BMI, may interfere with GH immunoassays, potentially leading to an underestimation of peak GH values [12,13,14,15,16,17].

Regardless of the underlying pathophysiological mechanisms, the inverse association between BMI and peak GH levels may lead to an overdiagnosis of GHD in children with higher BMI, increasing the risk of false-positive test results. Therefore, the aim of the present study was to further examine the association between BMI and peak GH response in children undergoing GH stimulation testing for suspected GHD, with particular focus on whether this association differs between patients ultimately diagnosed with GHD and those with short stature but normal GH secretion. Given previous reports of this association in these clinical groups, our study was intended to provide additional evidence and a direct comparison of the relationship between BMI and GH response across diagnostic categories.

## 2. Materials and Methods

### 2.1. Study Population

We conducted a single-center retrospective observational study including 298 children referred to the Pediatric Endocrinology Unit of the Fondazione Policlinico Universitario Agostino Gemelli IRCCS (Rome, Italy) between January 2021 and May 2026 for the evaluation of short stature and/or growth failure. All participants underwent GH stimulation testing as part of the diagnostic work-up for suspected GHD. Exclusion criteria were: (1) conditions known to affect BMI, including Cushing’s syndrome, hypothyroidism, and hypothalamic obesity; (2) treatment with medications known to influence BMI; (3) chronic systemic diseases, genetic syndromes, or other disorders affecting growth, including celiac disease; and (4) incomplete clinical data. Clinical records were retrospectively reviewed, and demographic, auxological, and biochemical data were collected. The variables analyzed included sex, age at testing, pubertal status, height, height SDS, weight, weight SDS, BMI and BMI SDS, IGF-1 and IGF-1 SDS, type of GH stimulation test (arginine or clonidine), and peak stimulated GH concentration.

### 2.2. Anthropometric Assessments

Height and weight were assessed according to standard clinical procedures. Height was measured by the same pediatric endocrinologist using a wall-mounted stadiometer, with subjects standing barefoot in the Frankfurt plane. Weight was measured using a calibrated electronic scale. BMI was calculated as weight (kg) divided by height squared (m^2^). Height, weight, and BMI SDS were calculated using the Italian reference data, according to the Italian Society of Pediatric Endocrinology and Diabetology (SIEDP) Guidelines [18]. IGF-1 SDS were calculated using the age- and sex-specific reference data provided for the assay used in our laboratory. Weight status was categorized based on BMI SDS as follows: normal weight (−2 SDS to +1 SDS), overweight (>+1 SDS), and obesity (>+2 SDS). Pubertal status was assessed according to Tanner staging. Puberty was outlined as breast development (Tanner stage B2 or higher) in girls and a testicular volume ≥4 mL in boys.

### 2.3. Biochemical Measurements

Serum GH concentrations were quantified using a chemiluminescent immunometric assay routinely employed in the hospital laboratory. IGF-1 concentrations were measured using standardized immunoassays and expressed both as absolute values and age- and sex-adjusted SDS.

### 2.4. Growth Hormone Stimulation Testing

GH secretion was evaluated using pharmacological stimulation tests with either arginine or clonidine, performed according to institutional protocols after an overnight fast. Arginine hydrochloride was administered intravenously at a dose of 0.5 g/kg body weight (maximum dose 30 g) over 30 min. Blood samples for GH determination were collected at baseline (0 min) and at 30, 60, 90, and 120 min after the start of the infusion. Clonidine was dispensed orally at a dose of 0.15 mg/m^2^ body surface area (maximum dose 0.25 mg). Blood pressure and heart rate were monitored throughout the procedure. Blood samples for GH measurement were collected at baseline (0 min) and at 30, 60, 90, and 120 min after drug administration. In both cases, the highest GH concentration measured during the test was considered the GH peak. A GH stimulation test was considered pathological when the stimulated GH peak was <8 ng/mL, according to the SIEDP Guidelines [18]. GHD was diagnosed according to institutional criteria requiring an inadequate GH response on two separate stimulation tests. For the purposes of the present study, the GH peak obtained during the stimulation test performed at the time of the study evaluation was analyzed in relation to BMI SDS. In patients with pathological tests, GHD diagnosis was subsequently confirmed by a second independent GH stimulation test. Sex-steroid priming was not routinely applied before GH stimulation testing, in accordance with local protocols.

### 2.5. Ethics Statement

According to the General Authorization for the Processing of Personal Data for Scientific Research Purposes (Authorization No. 9/2014), retrospective studies do not require approval from an ethics committee when identification codes are used to ensure that patients’ data cannot be traced back to them.

### 2.6. Statistical Analysis

Continuous variables are presented as mean ± standard deviation (SD) or median (interquartile range [IQR]), according to their distribution, whereas categorical variables are expressed as frequencies and percentages. Comparisons between patients with and without GHD were performed using Student’s *t*-test or the Mann–Whitney U test, as appropriate, while categorical variables were compared using Pearson’s χ^2^ test. The association between BMI SDS and stimulated GH peak was initially assessed using Pearson’s and Spearman’s correlation coefficients. Correlation analyses were conducted in the overall cohort and in predefined subgroups according to GHD status, sex, pubertal status, and GH stimulation test (arginine or clonidine). Differences in correlation coefficients between subgroups were evaluated using Fisher’s z transformation. The primary analysis consisted of a multivariable linear regression model evaluating the association between BMI SDS and stimulated GH peak after adjustment for age, sex, pubertal status. Regression coefficients (β) with 95% confidence intervals (95% CIs) and corresponding *p* values were reported. To identify independent predictors of GHD, multivariable logistic regression analysis was performed using BMI SDS as the main exposure variable and adjusting for age, sex, and pubertal status. Results are presented as odds ratios (ORs) with 95% confidence intervals (95% CIs). To assess the robustness of the findings while limiting the number of covariates in the primary models, sensitivity analyses were performed by additionally adjusting both models for IGF-1 SDS and height SDS. As a secondary analysis, patients were stratified into quartiles according to BMI SDS. Differences in stimulated GH peak and GHD prevalence across quartiles were evaluated, and linear and logistic regression models including BMI SDS quartiles as an ordinal variable were fitted to assess linear trends across increasing BMI SDS categories (P for trend). All statistical tests were two-sided, and a *p* value < 0.05 was considered statistically significant. As this was a retrospective study including all eligible patients during the predefined study period, no a priori sample-size calculation was performed. A post hoc power analysis was therefore conducted based on the observed Pearson correlation coefficients between BMI SDS and stimulated GH peak, using a two-sided α level of 0.05. The achieved statistical power was 99.96% for the overall cohort, 94.0% for the GHD subgroup, and 28.0% for the non-GHD subgroup. Finally, as an exploratory analysis, BMI-specific GH peak cut-offs were derived within each BMI SDS quartile. Receiver operating characteristic (ROC) curve analyses were performed separately in the four BMI SDS quartiles using confirmed GHD diagnosis as the outcome. Within each quartile, the GH peak threshold maximizing the Youden index (sensitivity + specificity − 1) was identified. The area under the ROC curve (AUC), sensitivity, and specificity at the selected threshold were calculated. Ninety-five percent confidence intervals for the AUC were estimated using 5000 bootstrap resamples. Given the exploratory, sample-derived nature of this analysis and the lack of an independent diagnostic reference standard, these thresholds were not considered validated diagnostic cut-offs.

## 3. Results

A total of 298 children evaluated for suspected GHD were included in the study. The cohort comprised 165 males (55.4%) and 133 females (44.6%). Overall, 221 children (74.2%) were prepubertal, while 77 (25.8%) had entered puberty. GHD was confirmed in 114 patients (38.3%). GH stimulation testing was performed using arginine in 151 patients (50.7%) and clonidine in 147 patients (49.3%). The main characteristics of the study population are summarized in Table 1.

### 3.1. Association Between BMI-SDS and Stimulated GH Peak

BMI SDS was inversely correlated with GH peak. Pearson correlation analysis showed a significant negative correlation (r = −0.299, *p* < 0.001), which was confirmed by Spearman rank correlation (ρ = −0.318, *p* < 0.001), as reported in Figure 1.

Each 1-SDS increase in BMI was independently associated with a 1.63 ng/mL decrease in GH peak after adjustment for age, sex and pubertal status (β = −1.63, 95% CI −2.20 to −1.07; *p* < 0.001), as reported in Table 2.

In a sensitivity analysis additionally adjusting for IGF-1 SDS and height SDS, the association between BMI SDS and stimulated GH peak remained significant and of similar magnitude (β = −1.80 ng/mL per 1-SDS increase, 95% CI −2.36 to −1.24; *p* < 0.001), supporting the robustness of the primary model (these results are described in the Supplementary Materials). Another sensitivity analysis using absolute BMI instead of BMI SDS yielded similar results, confirming the robustness of the observed associations. In the overall cohort, BMI was significantly and inversely correlated with stimulated GH peak (Spearman’s r = −0.289, *p* = 3.8 × 10^−7^; Pearson’s r = −0.263, *p* = 4.4 × 10^−6^), indicating that increasing BMI was associated with lower GH responses during stimulation testing, as reported in Figure 2.

In the primary analysis using BMI SDS, when patients were stratified according to GHD status, no significant correlation was observed among patients without GHD (Spearman’s r = −0.073, *p* = 0.327), whereas a significant inverse association was found in patients with GHD (Spearman’s r = −0.336, *p* < 0.001). The inverse association between BMI SDS and GH peak was similar in males (Spearman’s r = −0.346, *p* < 0.001) and females (Spearman’s r = −0.331, *p* < 0.001). When stratified according to pubertal status, the association was stronger in pubertal subjects (r = −0.483, *p* < 0.001) than in prepubertal children (r = −0.256, *p* < 0.001). Comparable results were obtained regardless of the stimulation test used, with similar correlation coefficients for clonidine (r = −0.307, *p* < 0.001) and arginine (r = −0.332, *p* < 0.001). These results are illustrated in the Supplementary Materials. Moreover, in the multivariable logistic regression model including BMI SDS, age, sex, and pubertal status, each 1-SDS increase in BMI was independently associated with a 2.10-fold higher odds of GHD, as reported in Table 3.

Similarly, in a sensitivity analysis additionally adjusted for IGF-1 SDS and height SDS, BMI SDS remained independently associated with GHD diagnosis (OR 2.29 per 1-SDS increase, 95% CI 1.73–3.03; *p* < 0.001), further supporting the robustness of the primary analysis (data shown in the Supplementary Materials). The inverse correlation between BMI SDS and GH peak was significant in the overall cohort and in all examined subgroups except patients without GHD. However, Fisher’s z transformation showed no statistically significant differences in the strength of the correlation between males and females (*p* = 0.682), between prepubertal and pubertal subjects (*p* = 0.112), or between arginine- and clonidine-stimulated patients (*p* = 0.983). Interestingly Fisher’s transformation showed a statistically significant difference in strength of the correlation between patients with and without GHD (*p* = 0.05). The main results are reported in Table 4.

### 3.2. BMI Quartile Analysis

Patients were stratified into quartiles according to BMI SDS, as reported in Table 5. A progressive decline in GH peak was observed across increasing BMI SDS quartiles (11.14, 8.79, 8.73 and 6.93 ng/mL, respectively; Kruskal–Wallis *p* < 0.0001). Likewise, the prevalence of confirmed GHD increased from 22.4% in the lowest BMI SDS quartile to 62.7% in the highest quartile (χ^2^ *p* < 0.0001), supporting a dose–response relationship between increasing BMI SDS and impaired GH secretion. Linear and logistic regression analyses treating BMI SDS quartiles as an ordinal variable demonstrated a significant inverse linear trend in GH peak and a progressive increase in GHD prevalence across increasing BMI SDS quartiles. Each one-quartile increase in BMI SDS was associated with a 1.27 ng/mL decrease in GH peak (β = −1.27 ng/mL per quartile; P for trend = 2.83 × 10^−6^) and a 76% increase in the odds of GHD (OR 1.76; P for trend < 0.001).

### 3.3. Exploratory BMI-Specific GH Cut-Offs

In an exploratory ROC analysis stratified by BMI SDS quartiles, the sample-derived GH peak thresholds maximizing the Youden index were 7.80 ng/mL in Q1 (BMI SDS −3.56 to −0.98), 7.00 ng/mL in Q2 (−0.94 to −0.18), 7.60 ng/mL in Q3 (−0.17 to 0.62), and 5.99 ng/mL in Q4 (0.63 to 2.10). The corresponding AUCs were 0.944, 0.900, 0.926, and 0.959, respectively. Notably, while the empirically derived thresholds were relatively similar across the first three BMI SDS quartiles, a substantially lower threshold of approximately 6 ng/mL was identified in the highest BMI SDS quartile. Full results, including sensitivity and specificity, are reported in the Supplementary Materials.

## 4. Discussion

Our retrospective study, including 298 children who underwent GH stimulation testing for suspected GHD, confirmed the inverse relationship between BMI SDS and stimulated GH peak [19,20,21,22,23]. Several studies have reported that increasing BMI SDS is related to lower GH responses during pharmacological stimulation tests. A recent meta-analysis by Abawi et al., including 58 studies and 5.135 children, confirmed a significant inverse association between BMI and peak GH response, showing that each 1-SDS increase in BMI was associated with an approximately 11.6% reduction in peak GH [12]. Importantly, this association was observed across the entire BMI spectrum, including children within the normal BMI range, suggesting that BMI influences GH responsiveness as a continuous biological variable rather than only in children with overweight or obesity [12]. Similarly, Ozdemir Dilek et al. reported progressively lower GH peak values with increasing BMI, from 8.2 ng/mL in normal-weight children to 5.9 ng/mL in those with overweight and 3.6 ng/mL in those with obesity [23]. Moreover, overweight and obese children showed lower test specificity and diagnostic accuracy compared with their normal-weight peers, highlighting the potential risk of overdiagnosing GHD in children with higher BMI and supporting the need to consider body composition when interpreting GH stimulation tests [23,24].

The main finding of our study was that the association between BMI SDS and GH peak was statistically significant in the GHD subgroup, whereas no statistically significant association was observed in the non-GHD subgroup.

These findings should be interpreted within the broader context of the existing body of evidence. Lee et al. specifically investigated short children without GHD and documented a significant inverse association between BMI SDS and peak stimulated GH, with BMI SDS being an independent predictor of peak GH, particularly in response to clonidine [13]. Thus, the absence of statistical significance in our non-GHD group contrasts with the findings of Lee et al. and does not establish that the BMI–GH relationship is restricted to children with GHD. Our findings are also broadly consistent with those of Lee et al., who evaluated children with short stature including both GHD and non-GHD and found an overall negative association between BMI SDS and peak stimulated GH [24]. In their study, BMI SDS emerged as an independent predictor of peak GH in the overall cohort, with subgroup analyses indicating a stronger association among children with GHD compared with those without GHD. Differences between studies may relate to sample characteristics, diagnostic criteria, stimulation protocols, and subgroup sample sizes. Taken together, the available evidence supports an inverse relationship between adiposity and stimulated GH response in children evaluated for short stature, while the strength and statistical significance of this association may vary across populations and GH secretory status. Importantly, differences in statistical significance between subgroups should not be interpreted as evidence of a statistically significant difference in the association between groups. Therefore, our subgroup findings should be considered hypothesis-generating rather than evidence that adiposity preferentially affects GH responses in children with GHD. Our findings support the need for further studies specifically designed to determine whether the association between BMI and GH response truly differs according to GH secretory status. The mechanisms underlying the relationship between adiposity and GH secretion are complex and involve multiple metabolic and hypothalamic pathways. Adiposity is a major modulator of the somatotropic axis and is associated with reduced spontaneous and stimulated GH secretion [25,26,27,28]. Several studies have demonstrated an inverse relationship between adiposity, particularly visceral fat accumulation, and GH pulsatile secretion, with obesity reducing GH pulse amplitude and overall daily GH output [25,26,27,28]. Veldhuis et al. showed that increased adiposity is strongly associated with decreased 24-h GH secretion, mainly through a reduction in the amplitude of GH secretory bursts. Importantly, this effect appears to involve alterations in GH secretion rather than simply a reduction in the frequency of secretory pulses. Proposed mechanisms include increased hypothalamic somatostatin tone, reduced GHRH drive, impaired ghrelin signalling, and metabolic alterations such as insulin resistance and increased circulating free fatty acids (FFAs) [27,28,29]. Elevated FFAs may directly suppress GH secretion, while hyperinsulinemia and insulin resistance may further modify the regulation of the GH/IGF-1 axis. In addition, visceral adiposity may contribute to this phenomenon through its metabolic activity and its association with systemic insulin resistance and altered adipokine signaling [27,28,29].

These mechanisms may be particularly relevant when interpreting stimulated GH responses in children with GHD. In the presence of impaired GH secretory capacity, the additional suppressive effects associated with increased adiposity could contribute to lower GH peaks during stimulation testing. This may provide a possible biological context for the inverse association between BMI SDS and stimulated peak GH observed in our GHD group. In our cohort, each 1-SDS increase in BMI was associated with a 1.63 ng/mL lower stimulated peak GH (β = −1.63 ng/mL/SDS), a finding directionally consistent with previous reports describing a reduction in stimulated GH with increasing adiposity. However, the mechanisms described above remain hypothetical in our study, as we did not assess insulin resistance, circulating FFAs, ghrelin, adipokines, or other metabolic or hypothalamic mediators. Moreover, the presence of a statistically significant association in the GHD group, in the absence of statistical significance in the non-GHD group, should not be interpreted as evidence that adiposity preferentially affects an already impaired somatotropic axis. Rather, our findings indicate that adiposity is associated with stimulated GH responses in children with confirmed GHD, while the extent to which this relationship differs according to GH secretory status remains to be established.

Interestingly, BMI SDS remained independently associated with stimulated GH peak after adjustment for age, sex, and pubertal status. Moreover, BMI SDS was independently associated with the diagnosis of GHD, with each 1-SDS increase in BMI SDS associated with 2.10-fold higher odds of GHD diagnosis (OR 2.10, 95% CI 1.60–2.74). These findings should be interpreted cautiously, as the relationship between BMI and GHD is bidirectional. On one hand, increased adiposity may contribute to reduced GH responsiveness through functional suppression of the somatotropic axis. On the other hand, GH deficiency itself promotes increased fat accumulation due to impaired lipolysis and alterations in body composition [29,30]. Therefore, the association observed in our study likely reflects a complex interaction between the inhibitory effect of adiposity on GH secretion and the metabolic consequences of GH deficiency. The dose–response relationship observed in our BMI SDS quartile analysis further supports the biological relevance of this association. Children in the highest BMI quartile showed progressively lower stimulated GH peaks and a higher prevalence of GHD compared with those in the lowest quartile. This gradient suggests that the relationship between adiposity and GH secretion is continuous and not restricted to children with severe obesity. Importantly, the association between BMI SDS and GH peak was consistent across both stimulation tests used in our cohort, suggesting that this effect is not dependent on the specific pharmacological stimulus employed.

These findings have important implications for the interpretation of GH stimulation tests. Current diagnostic approaches generally rely on fixed GH cut-off values without routinely accounting for BMI, despite increasing evidence that adiposity influences GH responsiveness. Our data suggest that BMI may be relevant when interpreting stimulated GH responses, including in children with borderline GH responses; however, whether its impact differs according to GH secretory status remains uncertain. Therefore, BMI and body composition should be considered when interpreting GH stimulation tests, although whether BMI-adjusted diagnostic thresholds may improve diagnostic accuracy requires confirmation in prospective multicenter studies. These findings raise the possibility of considering BMI-adjusted peak GH cutoffs in children evaluated for suspected GHD. Abawi et al. proposed a weight-status-adjusted approach based on their individual participant data analysis, showing that peak GH decreased by approximately 11.6% for each one-point increase in BMI SDS [12]. Based on this relationship, they proposed adjusting the conventional peak GH cutoff according to BMI SDS, using the equation: adjusted cutoff = normal-weight cutoff × 0.929^BMI SDS. Thus, assuming a conventional cutoff of 10 µg/L for children with normal weight, the proposed cutoff would decrease to approximately 9.3 µg/L in children with overweight (BMI SDS ≥ 1) and 8.6 µg/L in those with obesity (BMI SDS ≥ 2). Similarly, if a cutoff of 7 µg/L is used for normal-weight children, the corresponding proposed thresholds would be 6.5 and 6.0 µg/L for overweight and obesity, respectively [12]. However, these BMI-adjusted cutoffs remain to be prospectively validated before clinical application. Our exploratory analysis of sample-derived BMI-specific thresholds provides additional support for the potential influence of adiposity on the interpretation of GH stimulation testing. While the optimal thresholds were relatively similar across the first three BMI SDS quartiles (7.0–7.8 ng/mL), the empirically derived threshold decreased to approximately 6 ng/mL in children in the highest BMI SDS quartile. This finding raises the possibility that the use of a fixed GH threshold may have a greater impact on diagnostic classification at higher BMI levels. However, these thresholds were derived from relatively small subgroups within the same cohort and GHD diagnosis was not based on a reference standard independent of GH stimulation testing. Therefore, these results should be regarded as hypothesis-generating and require prospective external validation before any clinical application.

## 5. Limitations

Several limitations should be acknowledged. First, the retrospective, single-center design may introduce selection bias and residual confounding and limit the generalizability of our findings to other pediatric populations. In particular, the lack of information on ethnic composition and the relatively specific study population may limit the representativeness of the findings across Italian and European populations. In addition, no a priori sample-size calculation was performed because the study was based on a retrospective cohort of all eligible patients during the predefined study period. Post hoc power analysis showed high statistical power for the observed association in the overall cohort and GHD subgroup, whereas power was limited in the non-GHD subgroup. Therefore, the absence of a statistically significant association in the non-GHD subgroup should be interpreted cautiously, as a weak association cannot be excluded.

Second, BMI SDS was used as a surrogate marker of adiposity and does not provide information on body composition or fat distribution. We also lacked data on potentially relevant metabolic factors, including insulin resistance and circulating free fatty acids, which may contribute to the relationship between adiposity and GH secretion. Third, only arginine and clonidine stimulation tests were included, and therefore our findings may not be generalizable to other pharmacological stimuli or stimulation protocols.

A further limitation relates to the classification of GHD based on stimulated GH peak values. Because subgroup allocation was defined according to the same GH response used in the correlation analyses, selection effects and restriction of the GH range within subgroups may have influenced the observed associations. In addition, the absence of a statistically significant association in the non-GHD group should not be interpreted as evidence that the BMI–GH relationship is specific to GHD or that adiposity preferentially affects an already impaired somatotropic axis. Finally, given the observational nature of the study, we cannot determine whether higher adiposity contributes to lower stimulated GH responses or whether other factors underlying GH status contribute to the observed association. Therefore, our findings should be interpreted as observational associations rather than evidence of a causal relationship.

## 6. Conclusions

Our study confirms an inverse association between BMI SDS and stimulated GH peak in children undergoing evaluation for suspected GHD. This association was statistically significant in children with confirmed GHD, while no statistically significant association was observed in children with short stature and preserved GH secretion. However, the absence of statistical significance in the latter group does not demonstrate that the association is exclusive to GHD or that adiposity preferentially affects an already impaired somatotropic axis. These findings highlight the potential relevance of adiposity when interpreting stimulated GH responses in children and support further investigation of BMI-aware approaches to GH stimulation testing. Prospective studies in larger cohorts are needed to determine whether and to what extent BMI-adjusted interpretation may improve the diagnostic evaluation of pediatric GHD.

## Figures and Tables

**Figure 1:**
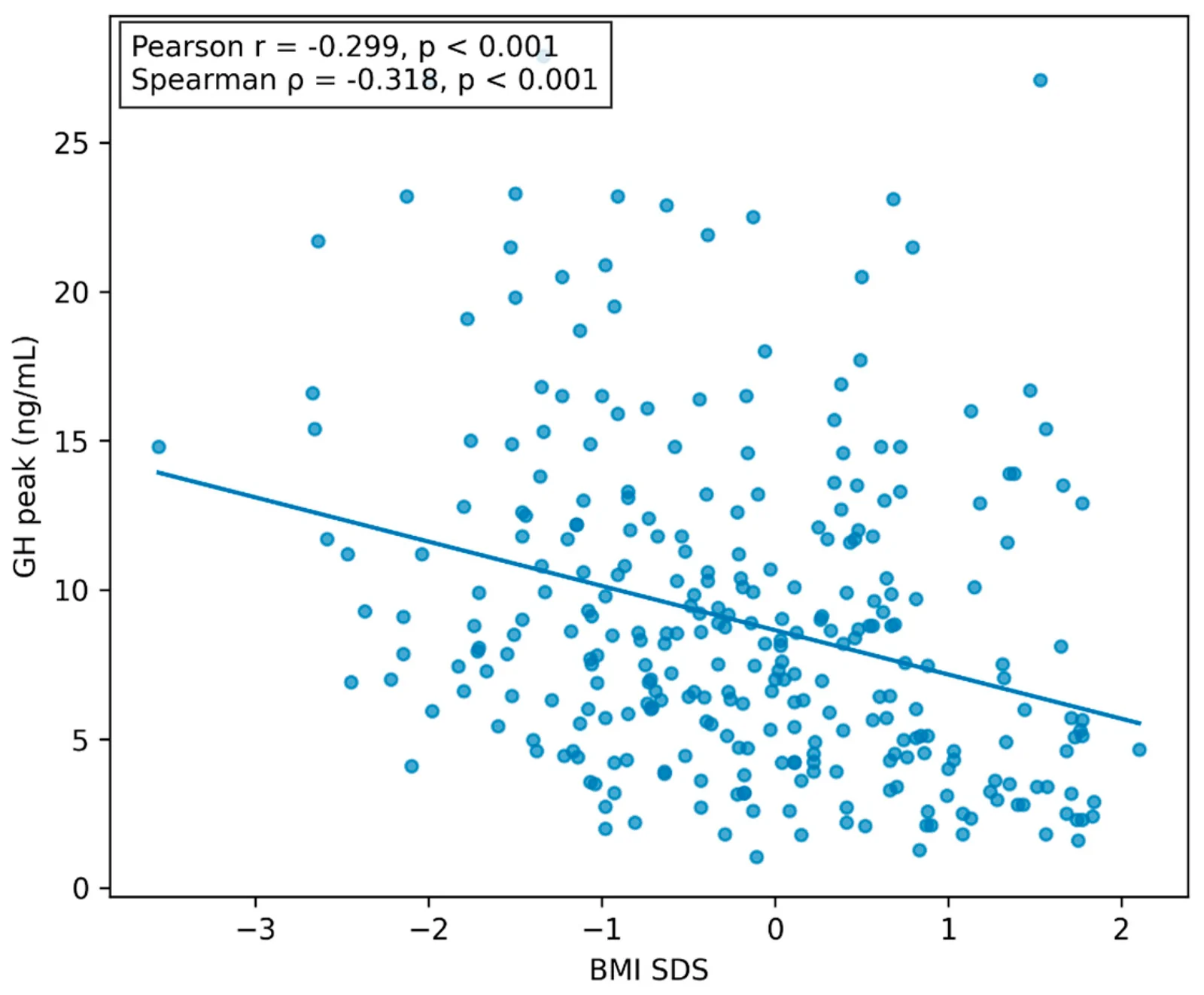
Correlation between GH peak and BMI-SDS found in our study.

**Figure 2:**
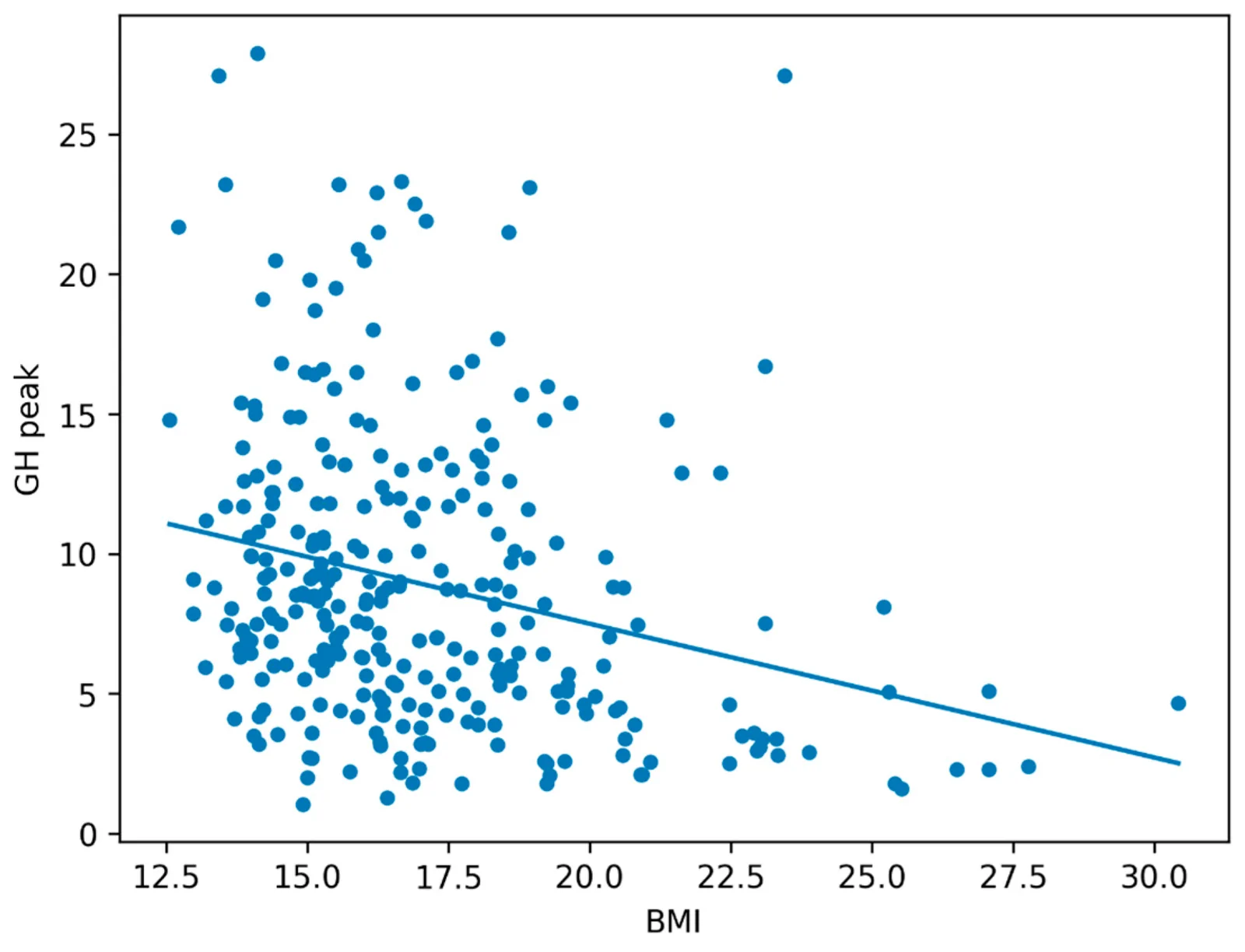
Correlation between GH peak and BMI found in our study.

**Table 1:** Baseline characteristics of the study population according to GHD status.

Variable	Overall (*n* = 298)	GHD (*n* = 114)	Non-GHD (*n* = 184)	*p* Value
Age, years †	9.50 ± 2.89	9.84 ± 2.93	9.30 ± 2.86	0.117
Height, m †	1.23 ± 0.16	1.25 ± 0.17	1.22 ± 0.16	0.106
Height SDS †	−2.02 ± 0.73	−1.98 ± 0.86	−2.05 ± 0.63	0.442
Weight, kg †	27.03 ± 10.46	30.06 ± 12.28	25.16 ± 8.67	<0.001
Weight SDS †	−1.36 ± 1.34	−1.01 ± 1.14	−1.57 ± 1.41	<0.001
BMI, kg/m^2^ †	17.06 ± 2.94	18.28 ± 3.52	16.30 ± 2.20	<0.001
BMI SDS †	−0.18 ± 1.08	0.25 ± 1.03	−0.44 ± 1.02	<0.001
IGF-1, ng/mL †	143.09 ± 78.60	137.80 ± 68.28	146.37 ± 84.38	0.338
IGF-1 SDS †	−1.59 ± 0.54	−1.67 ± 0.42	−1.54 ± 0.60	0.024
GH peak, ng/mL ‡	7.90 (4.76–11.80)	4.39 (3.00–6.22)	10.40 (8.46–14.07)	<0.001
Female sex, *n* (%) §	133 (44.6)	52 (45.6)	81 (44.0)	0.882
Prepubertal status, *n* (%) §	221 (74.2)	78 (68.4)	143 (77.7)	0.100

Data are presented as mean ± standard deviation (SD), median (interquartile range [IQR]), or number (percentage), as appropriate. † Student’s *t*-test. ‡ Mann–Whitney U test. § Pearson’s χ^2^ test. Abbreviations: BMI, body mass index; SDS, standard deviation score; GHD, growth hormone deficiency; GH, growth hormone; IGF-1, insulin-like growth factor-1.

**Table 2:** Multiple linear regression analysis of factors associated with peak GH levels.

Variable	β (ng/mL)	IC95%	*p*
BMI SDS	−1.63	−2.20 to −1.07	<0.001
Age (years)	+0.06	−0.21 to +0.32	0.679
Sex (female vs. male)	−1.27	−2.53 to +0.00	0.051
Prepubertal vs. pubertal	+0.69	−0.99 to +2.37	0.421

**Table 3:** Multivariable Logistic Regression Analysis of Factors Associated with GHD Diagnosis.

Variable	OR (IC95%)	*p*
BMI SDS	2.10 (1.60–2.74)	<0.001
Age (years)	1.07 (0.96–1.20)	0.234
Sex (female vs. male)	1.92 (1.10–3.36)	0.021
Prepubertal vs. pubertal	0.85 (0.42–1.73)	0.662

**Table 4:** Correlation between BMI SDS and GH peak in the overall cohort and according to clinical subgroups.

Subgroup	*n*	Pearson r	*p* Value	Spearman ρ	*p* Value
Overall	298	−0.299	<0.001	−0.318	<0.001
GHD	114	−0.322	<0.001	−0.336	<0.001
Non-GHD	184	−0.102	0.167	−0.073	0.327
Male	165	−0.301	<0.001	−0.346	<0.001
Female	133	−0.344	<0.001	−0.331	<0.001
Prepubertal	221	−0.244	<0.001	−0.256	<0.001
Pubertal	77	−0.432	<0.001	−0.483	<0.001
Arginine stimulation test	151	−0.303	<0.001	−0.332	<0.001
Clonidine stimulation test	147	−0.301	<0.001	−0.307	<0.001
Fisher’s z comparison of Pearson correlation coefficients					
Comparison	Fisher’s z				*p* value
GHD vs. non-GHD	−1.92				0.050
Male vs. female	0.41				0.682
Prepubertal vs. pubertal	1.59				0.112
Arginine vs. clonidine	−0.02				0.983

**Table 5:** Peak GH Levels and GHD Prevalence Stratified by BMI SDS Quartiles.

Quartile	*n*	BMI SDS	GH Peak (ng/mL)	GHD *n* (%)
Q1	76	−1.55 ± 0.53	11.14 ± 5.90	17 (22.4%)
Q2	74	−0.55 ± 0.24	8.79 ± 4.67	23 (31.1%)
Q3	73	0.22 ± 0.23	8.73 ± 4.60	27 (37.0%)
Q4	75	1.19 ± 0.41	6.93 ± 5.36	47 (62.7%)

## Data Availability

No new data were created or analyzed in this study. Data sharing is not applicable to this article.

## Supplementary Materials

The following supporting information can be downloaded at: https://www.mdpi.com/article/10.3390/children13101345/s1. S1. Association between BMI-SDS and stimulated GH peak. S2. Supplementary Table: IGF-1 SDS and Height SDS adjusted multivariable linear regression analysis of stimulated GH peak and GHD diagnosis. S3. Exploratory BMI-adjusted GH Peak Cut-off Analysis.

## Abbreviations

The following abbreviations are used in this manuscript:

BMI	Body Mass Index
CI	Confidence Interval
GH	Growth Hormone
GHBP	Growth Hormone-Binding Protein
GHD	Growth Hormone Deficiency
GHRH	Growth Hormone-Releasing Hormone
IGF-1	Insulin-like Growth Factor-1
IGFBP-1	Insulin-like Growth Factor-Binding Protein 1
IGFBP-3	Insulin-like Growth Factor-Binding Protein 3
IQR	Interquartile Range
OR	Odds Ratio
SD	Standard Deviation
SDS	Standard Deviation Score
SIEDP	Italian Society of Pediatric Endocrinology and Diabetology

## References

[1] Cammisa I., De Fortuna G., Rulli E., Rigante D., Cipolla C. (2026). Predictors of Response to Growth Hormone Therapy in Children with Idiopathic Growth Hormone Deficiency: A Narrative Review. Children.

[2] Stanley T. (2012). Diagnosis of growth hormone deficiency in childhood. Curr. Opin. Endocrinol. Diabetes Obes..

[3] Chinoy A., Murray P.G. (2016). Diagnosis of growth hormone deficiency in the paediatric and transitional age. Best. Pract. Res. Clin. Endocrinol. Metab..

[4] Richmond E.J., Rogol A.D. (2008). Growth hormone deficiency in children. Pituitary.

[5] Arzilli F., De Fortuna G., Cammisa I., Vagnozzi L., Sodero G., Rigante D., Cipolla C. (2025). Diagnostic Challenges of Short Stature and Growth Hormone Insufficiency Across Different Genetic Etiologies. Biomedicines.

[6] Ibba A., Corrias F., Guzzetti C., Casula L., Salerno M., di Iorgi N., Tornese G., Patti G., Radetti G., Maghnie M. (2020). IGF1 for the diagnosis of growth hormone deficiency in children and adolescents: A reappraisal. Endocr. Connect..

[7] Chesover A.D., Dattani M.T. (2016). Evaluation of growth hormone stimulation testing in children. Clin. Endocrinol..

[8] Grimberg A., DiVall S.A., Polychronakos C., Allen D.B., Cohen L.E., Quintos J.B., Rossi W.C., Feudtner C., Murad M.H. (2016). Drug and Therapeutics Committee and Ethics Committee of the Pediatric Endocrine Society. Guidelines for Growth Hormone and Insulin-Like Growth Factor-I Treatment in Children and Adolescents: Growth Hormone Deficiency, Idiopathic Short Stature, and Primary Insulin-Like Growth Factor-I Deficiency. Horm. Res. Paediatr..

[9] Duncan G., Kiff S., Mitchell R.T. (2023). Sex steroid priming for growth hormone stimulation testing in children and adolescents with short stature: A systematic review. Clin. Endocrinol..

[10] Collett-Solberg P.F., Ambler G., Backeljauw P.F., Bidlingmaier M., Biller B.M.K., Boguszewski M.C.S., Cheung P.T., Choong C.S.Y., Cohen L.E., Cohen P. (2019). Diagnosis, Genetics, and Therapy of Short Stature in Children: A Growth Hormone Research Society International Perspective. Horm. Res. Paediatr..

[11] Cammisa I., Rigante D., Sodero G., Cipolla C. (2025). Sex Steroid Priming on Growth Hormone Stimulation Test: A Scoping Review. Children.

[12] Abawi O., Augustijn D., Hoeks S.E., de Rijke Y.B., van den Akker E.L.T. (2021). Impact of body mass index on growth hormone stimulation tests in children and adolescents: A systematic review and meta-analysis. Crit. Rev. Clin. Lab. Sci..

[13] Lee J., Yoon J., Kang M.J., Lee Y.A., Lee S.Y., Shin C.H., Yang S.W. (2013). Influence of body mass index on the growth hormone response to provocative testing in short children without growth hormone deficiency. J. Korean Med. Sci..

[14] Yau M., Chacko E., Regelmann M.O., Annunziato R., Wallach E.J., Chia D., Rapaport R. (2019). Peak Growth Hormone Response to Combined Stimulation Test in 315 Children and Correlations with Metabolic Parameters. Horm. Res. Paediatr..

[15] Conover C.A., Lee P.D., Kanaley J.A., Clarkson J.T., Jensen M.D. (1992). Insulin regulation of insulin-like growth factor binding protein-1 in obese and nonobese humans. J. Clin. Endocrinol. Metab..

[16] Hansen T.K., Fisker S., Hansen B., Sørensen H.H., Christiansen J.S., Jørgensen J.O., Ørskov H. (2002). Impact of GHBP interference on estimates of GH and GH pharmacokinetics. Clin. Endocrinol..

[17] Bidlingmaier M., Strasburger C.J. (2007). Growth hormone assays: Current methodologies and their limitations. Pituitary.

[18] Italian Society of Pediatric Endocrinology and Diabetology (SIEDP) (2018). Clinical Practice Pathway for the Diagnosis and Management of Growth Hormone Deficiency in Children and Adolescents.

[19] Patel L., Skinner A.M., Price D.A., Clayton P.E. (1994). The influence of body mass index on growth hormone secretion in normal and short statured children. Growth Regul..

[20] Stanley T.L., Levitsky L.L., Grinspoon S.K., Misra M. (2009). Effect of body mass index on peak growth hormone response to provocative testing in children with short stature. J. Clin. Endocrinol. Metab..

[21] Barrett J., Maranda L., Nwosu B.U. (2014). The Relationship between Subnormal Peak-Stimulated Growth Hormone Levels and Auxological Characteristics in Obese Children. Front. Endocrinol..

[22] Yang A., Cho S.Y., Kwak M.J., Kim S.J., Park S.W., Jin D.K., Lee J.E. (2019). Impact of BMI on peak growth hormone responses to provocative tests and therapeutic outcome in children with growth hormone deficiency. Sci. Rep..

[23] Ozdemir Dilek S., Özgüç Comlek F. (2026). Obesity-Related Changes in Growth Hormone Stimulation Test Performance Under Pediatric Growth Hormone Deficiency. Children.

[24] Lee N.Y., Kim S.E., Kim S., Ahn M.B., Kim S.H., Cho W.K., Cho K.S., Jung M.H., Suh B.K. (2021). Effect of body mass index on peak growth hormone level after growth hormone stimulation test in children with short stature. Ann. Pediatr. Endocrinol. Metab..

[25] Veldhuis J.D., Hudson S.B., Erickson D., Bailey J.N., Reynolds G.A., Bowers C.Y. (2009). Relative effects of estrogen, age, and visceral fat on pulsatile growth hormone secretion in healthy women. Am. J. Physiol. Endocrinol. Metab..

[26] Veldhuis J.D., Erickson D., Mielke K., Farhy L.S., Keenan D.M., Bowers C.Y. (2005). Distinctive inhibitory mechanisms of age and relative visceral adiposity on growth hormone secretion in pre- and postmenopausal women studied under a hypogonadal clamp. J. Clin. Endocrinol. Metab..

[27] Veldhuis J.D., Iranmanesh A. (1996). Physiological regulation of the human growth hormone (GH)-insulin-like growth factor type I (IGF-I) axis: Predominant impact of age, obesity, gonadal function, and sleep. Sleep.

[28] Veldhuis J.D., Liem A.Y., South S., Weltman A., Weltman J., Clemmons D.A., Abbott R., Mulligan T., Johnson M.L., Pincus S. (1995). Differential impact of age, sex steroid hormones, and obesity on basal versus pulsatile growth hormone secretion in men as assessed in an ultrasensitive chemiluminescence assay. J. Clin. Endocrinol. Metab..

[29] Scacchi M., Orsini F., Cattaneo A., Grasso A., Filippini B., Pecori Giraldi F., Fatti L.M., Moro M., Cavagnini F. (2010). The diagnosis of GH deficiency in obese patients: A reappraisal with GHRH plus arginine testing after pharmacological blockade of lipolysis. Eur. J. Endocrinol..

[30] Sharma V.M. (2019). Emerging mechanisms of GH-induced lipolysis and insulin resistance. Pediatr. Endocrinol. Rev..

