# Supplementary material for: The Effect of Body Mass Index on Peak Growth Hormone Response During Stimulation Testing: A Retrospective Analysis
Source: Children (Basel). 2026 Oct 3;13(10):1345. doi: 10.3390/children13101345 (PMC13702870; doi:10.3390/children13101345)
Supplement: Supplementary file 1 [file children-13-01345-s001.zip › children-4566084-supplementary.pdf]

S1. Association between BMI-SDS and stimulated GH peak

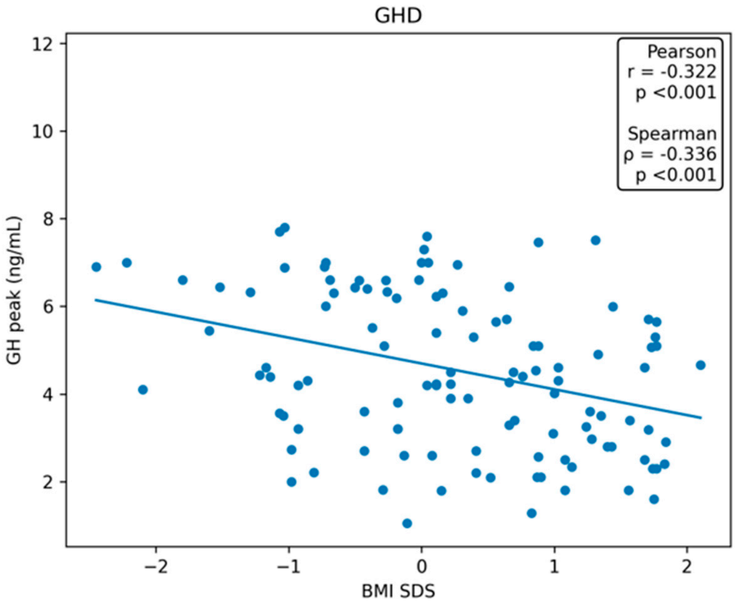

Figure S1. Association between BMI-SDS and stimulated GH peak in GHD children.

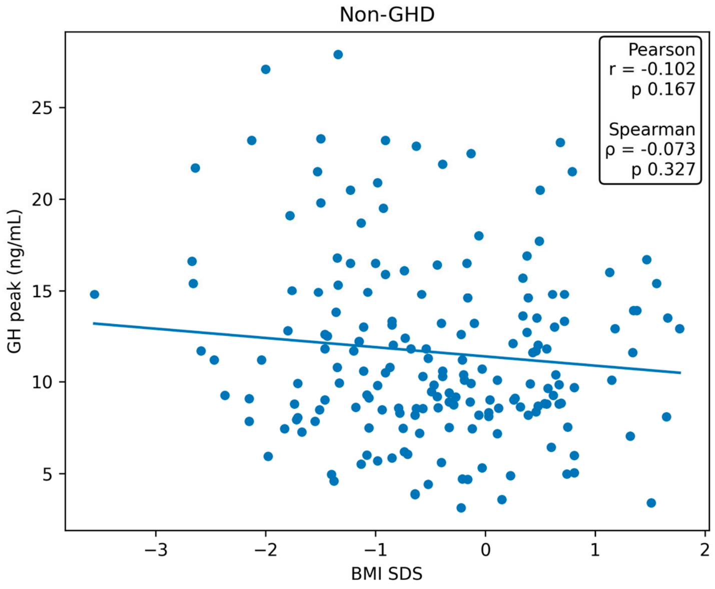

Figure S2. Association between BMI-SDS and stimulated GH peak in non-GHD children.

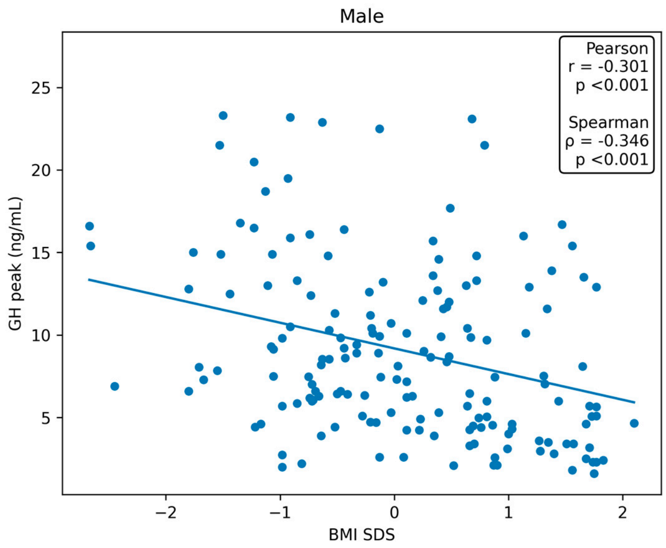

Figure S3. Association between BMI-SDS and stimulated GH peak in males.

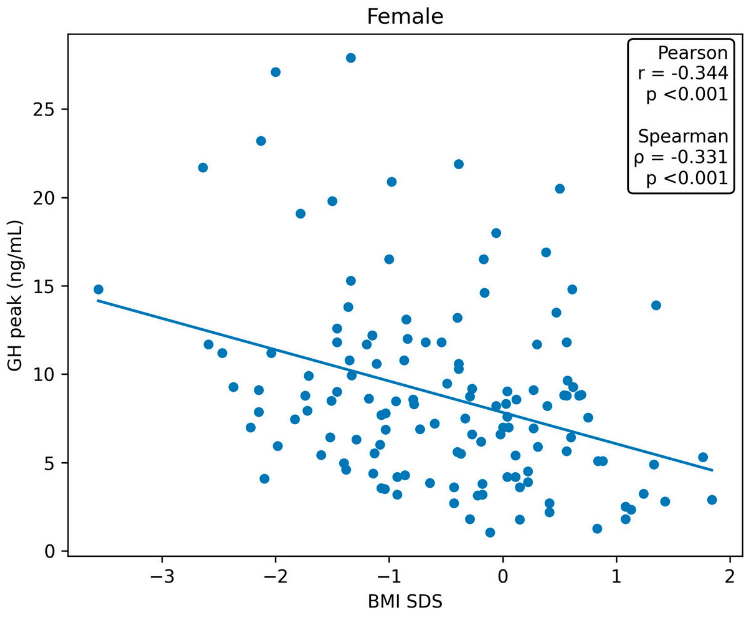

Figure S4. Association between BMI-SDS and stimulated GH peak in females.

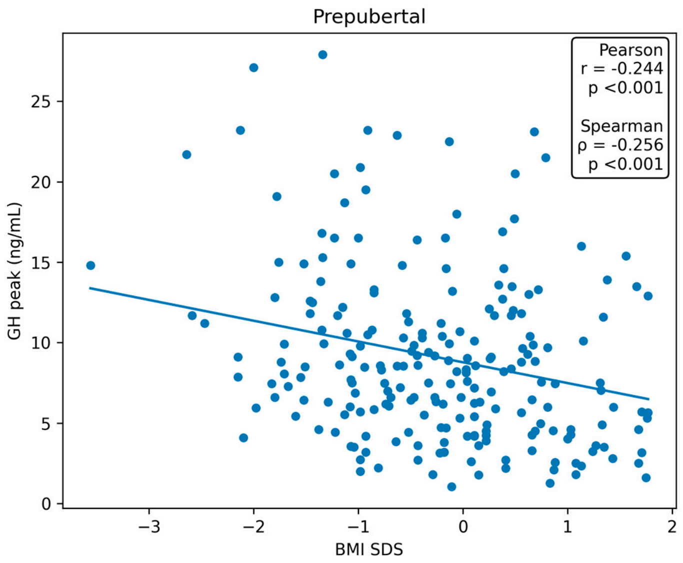

Figure S5. Association between BMI-SDS and stimulated GH peak in prepubertal children.

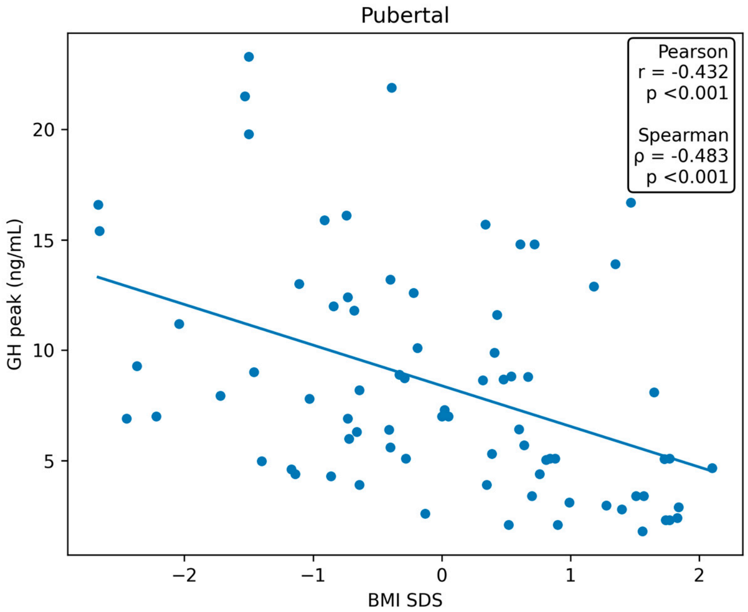

Figure S6. Association between BMI-SDS and stimulated GH peak in pubertal children.

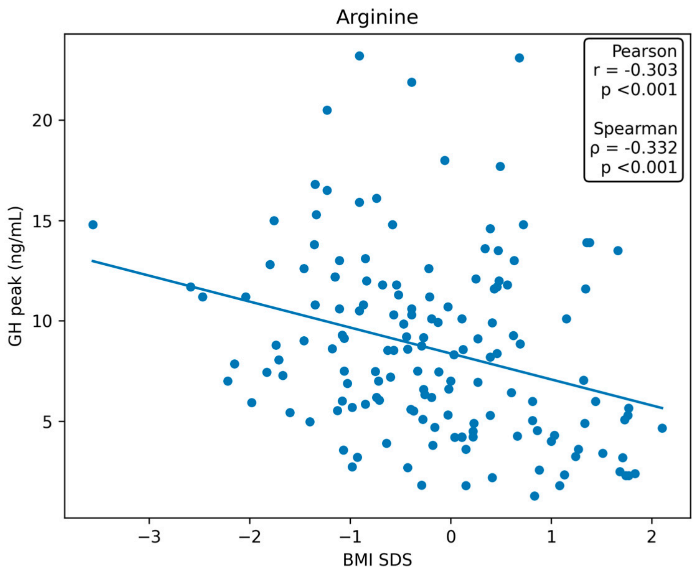

Figure S7. Association between BMI-SDS and stimulated GH peak in arginine tests.

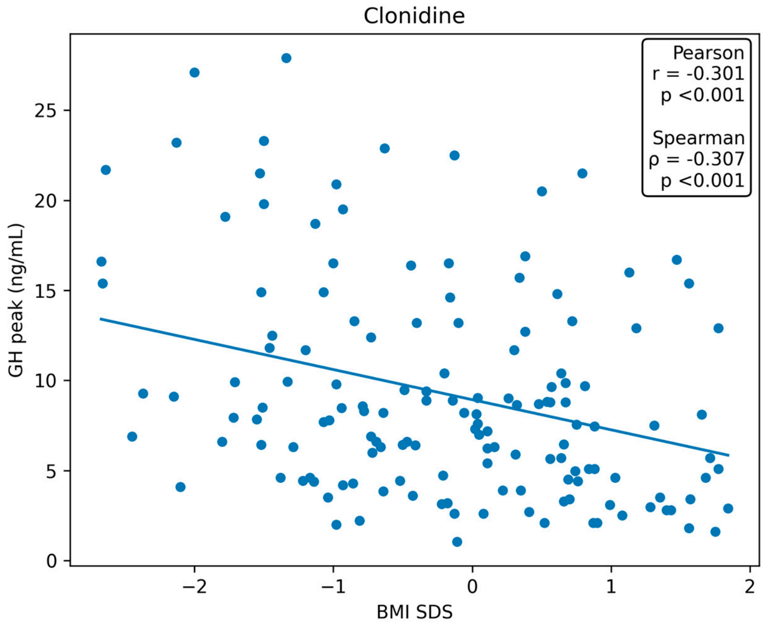

Figure S8. Association between BMI-SDS and stimulated GH peak in clonidine tests.

S2. upplementary Table: IGF-1 SDS and Height SDS adjusted multivariable linear regression analysis of stimulated GH peak and GHD diagnosis

Sensitivity analysis

Supplementary Table S1: IGF-1 SDS and height SDS adjusted multivariable linear regression analysis of stimulated GH peak.

Variable	β (ng/mL)	95% CI	p value
BMI SDS	−1.80	−2.36 to −1.24	<0.001
Age (years)	0.07	−0.19 to 0.32	0.615
Female vs male	−0.67	−1.96 to 0.63	0.313
Prepubertal vs pubertal	0.72	−0.94 to 2.38	0.394
IGF-1 SDS	2.10	0.96 to 3.24	<0.001
Height SDS	−0.12	−0.95 to 0.72	0.785

Model: stimulated GH peak as dependent variable; BMI SDS, age, sex, pubertal status, IGF-1 SDS, and height SDS as independent variables. N = 298. R^2 = 0.143; adjusted R^2 = 0.126. β values are unstandardized regression coefficients. CI, confidence interval; GH, growth hormone; IGF-1, insulin-like growth factor 1; SDS, standard deviation score.

Supplementary Table S2: IGF-1 SDS and height SDS adjusted multivariable logistic regression analysis of GHD diagnosis.

Variable	OR	95% CI	p value
BMI SDS	2.29	1.73 to 3.03	<0.001
Age (years)	1.06	0.95 to 1.19	0.302
Female vs male	1.55	0.87 to 2.75	0.139
Prepubertal vs pubertal	0.83	0.40 to 1.73	0.615
IGF-1 SDS	0.41	0.24 to 0.71	0.001
Height SDS	1.13	0.79 to 1.61	0.499

Model: GHD diagnosis as dependent variable; BMI SDS, age, sex, pubertal status, IGF-1 SDS, and height SDS as independent variables. N = 298 (114 GHD and 184 non-GHD). ORs are

reported with 95% confidence intervals. CI, confidence interval; GHD, growth hormone deficiency; IGF-1, insulin-like growth factor 1; OR, odds ratio; SDS, standard deviation score.

S3. Exploratory BMI-adjusted GH Peak Cut-off Analysis

Methods

Participants were divided into four quartiles according to BMI SDS. Within each quartile, receiver operating characteristic (ROC) analysis was performed using confirmed GHD diagnosis as the outcome and stimulated GH peak as the classifier, with lower GH peak values indicating GHD. The sample-derived GH peak threshold maximizing the Youden index (sensitivity + specificity - 1) was selected. AUC 95% confidence intervals were estimated using 5,000 bootstrap resamples. Exact binomial (Clopper–Pearson) 95% confidence intervals were calculated for sensitivity and specificity. Positive and negative predictive values, accuracy, positive and negative likelihood ratios, and confusion-matrix counts were also calculated. These analyses are exploratory and the derived thresholds are not externally validated.

Table S1. Characteristics of BMI SDS quartiles.

Quartile	N	BMI SDS range	BMI SDS mean ± SD	GH peak mean ± SD	GH peak median	GHD n (%)	Non-GHD n (%)
Q1	76	-3.56 to -0.98	-1.55 ± 0.53	11.14 ± 5.90	9.55	17 (22.4%)	59 (77.6%)
Q2	74	-0.94 to -0.18	-0.55 ± 0.24	8.79 ± 4.67	8.39	23 (31.1%)	51 (68.9%)
Q3	73	-0.17 to 0.62	0.22 ± 0.23	8.73 ± 4.60	8.32	27 (37.0%)	46 (63.0%)
Q4	75	0.63 to 2.10	1.19 ± 0.41	6.93 ± 5.36	5.04	47 (62.7%)	28 (37.3%)

Table S2. Sample-derived BMI-specific GH peak cut-offs and ROC performance.

Quartile	Cut-off	Youden	AUC (95% CI)	Sensitivity (95% CI)	Specificity (95% CI)	PPV	NPV	Accuracy	LR+	LR-
Q1	7.80	0.847	0.944 (0.887-0.984)	100.0% (80.5-100.0)	84.7% (73.0-92.8)	65.4%	100.0%	88.2%	6.56	0.00
Q2	7.00	0.824	0.900 (0.826-0.962)	100.0% (85.2-100.0)	82.4% (69.1-91.6)	71.9%	100.0%	87.8%	5.67	0.00
Q3	7.60	0.811	0.926 (0.855-0.981)	96.3% (81.0-99.9)	84.8% (71.1-93.7)	78.8%	97.5%	89.0%	6.33	0.04
Q4	5.99	0.829	0.959 (0.903-0.997)	93.6% (82.5-98.7)	89.3% (71.8-97.7)	93.6%	89.3%	92.0%	8.74	0.07

Table S3. Classification counts at the Youden-optimal threshold.

Quartile	Cut-off	True positive	False negative	True negative	False positive
Q1	7.80	17	0	50	9
Q2	7.00	23	0	42	9
Q3	7.60	26	1	39	7
Q4	5.99	44	3	25	3

Figure S1. ROC curves by BMI SDS quartile.

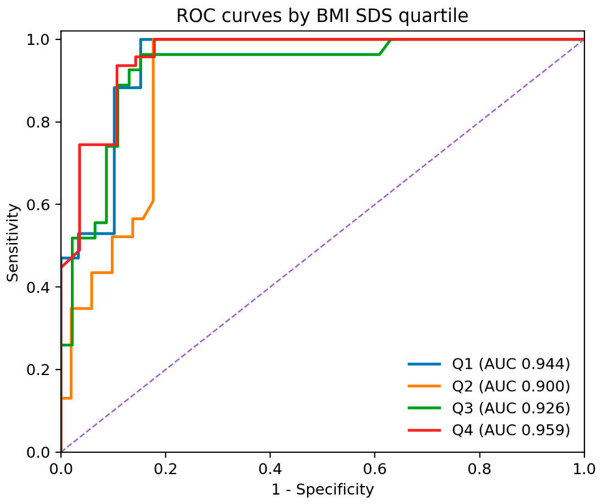

ROC curves for stimulated GH peak as a classifier of confirmed GHD, stratified by BMI SDS quartile.

Figure S2. ROC curve for Q1.

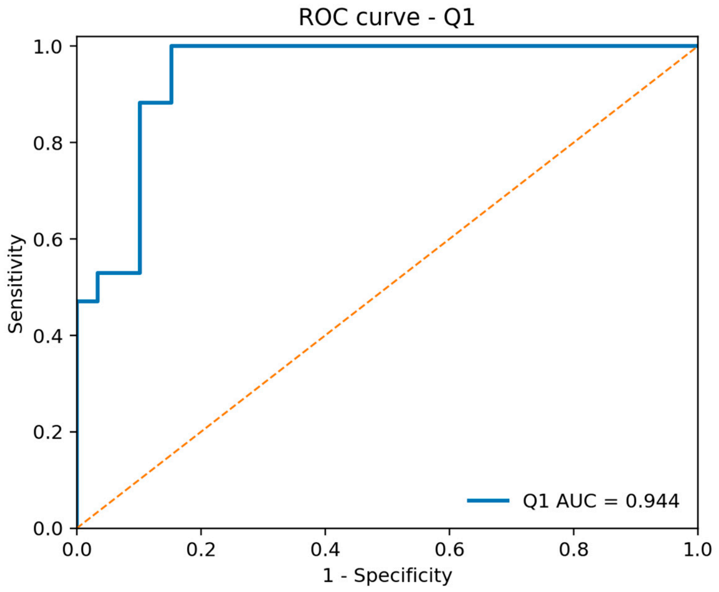

Q1: BMI SDS -3.56 to -0.98; n=76; AUC=0.944 (95% CI 0.887-0.984); Youden-optimal GH peak cut-off=7.80 ng/mL.

Figure S3. ROC curve for Q2.

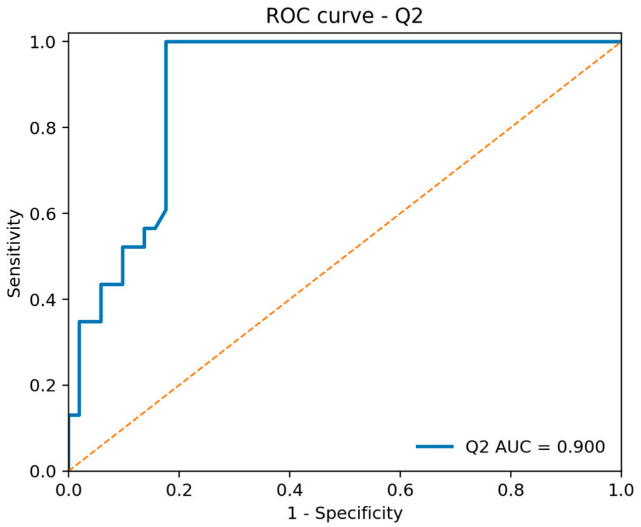

Q2: BMI SDS -0.94 to -0.18; n=74; AUC=0.900 (95% CI 0.826-0.962); Youden-optimal GH peak cut-off=7.00 ng/mL.

Figure S4. ROC curve for Q3.

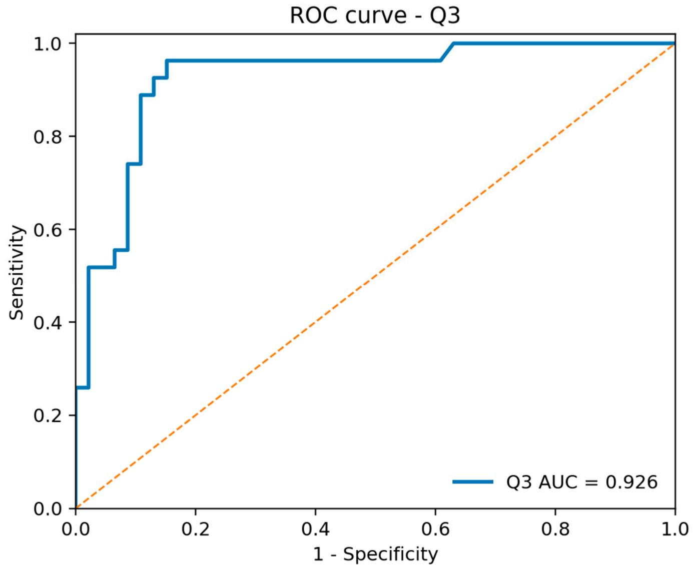

Q3: BMI SDS -0.17 to 0.62; n=73; AUC=0.926 (95% CI 0.855-0.981); Youden-optimal GH peak cut-off=7.60 ng/mL.

Figure S5. ROC curve for Q4.

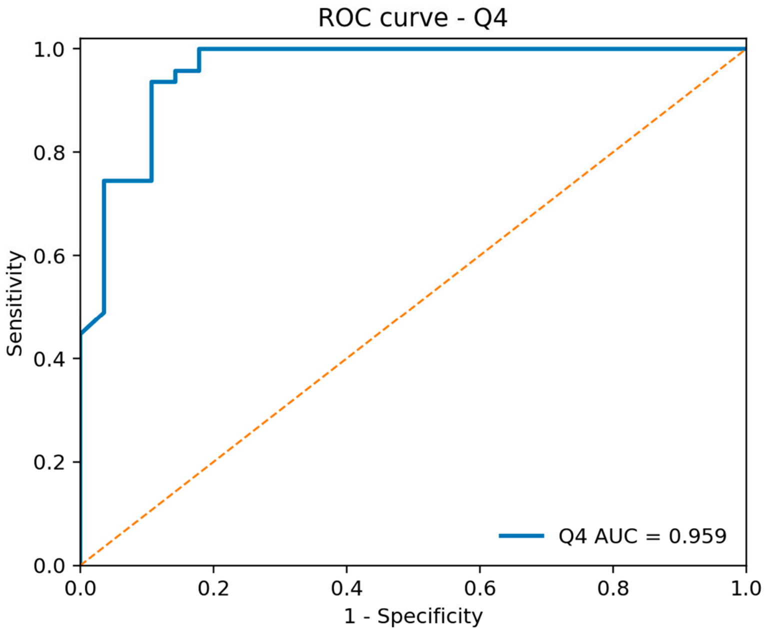

Q4: BMI SDS 0.63 to 2.10; n=75; AUC=0.959 (95% CI 0.903-0.997); Youden-optimal GH peak cut-off=5.99 ng/mL.

Complete ROC coordinates

The following tables report every empirical ROC threshold evaluated within each BMI SDS quartile. The selected Youden-optimal threshold is identified in the final column.

Table S4(1). Full ROC coordinates—Q1.

GH cut-off (ng/mL)	Sensitivity	Specificity	1 - Specificity	Youden index	Status
2.000	5.9%	100.0%	0.0%	0.059	
4.600	47.1%	100.0%	0.0%	0.471	
4.970	47.1%	96.6%	3.4%	0.437	
5.440	52.9%	96.6%	3.4%	0.496	
6.010	52.9%	89.8%	10.2%	0.428	
7.000	88.2%	89.8%	10.2%	0.781	
7.500	88.2%	84.7%	15.3%	0.730	
7.800	100.0%	84.7%	15.3%	0.847	Selected
10.800	100.0%	55.9%	44.1%	0.559	
11.700	100.0%	49.2%	50.8%	0.492	
11.800	100.0%	47.5%	52.5%	0.475	
12.200	100.0%	44.1%	55.9%	0.441	
14.800	100.0%	33.9%	66.1%	0.339	
14.900	100.0%	30.5%	69.5%	0.305	
15.400	100.0%	25.4%	74.6%	0.254	
16.500	100.0%	22.0%	78.0%	0.220	
27.900	100.0%	0.0%	100.0%	0.000	

Table S4(2). Full ROC coordinates—Q2.

GH cut-off (ng/mL)	Sensitivity	Specificity	1 - Specificity	Youden index	Status
1.810	4.3%	100.0%	0.0%	0.043	
2.700	13.0%	100.0%	0.0%	0.130	
3.140	13.0%	98.0%	2.0%	0.111	
3.200	26.1%	98.0%	2.0%	0.241	
3.800	34.8%	98.0%	2.0%	0.328	
3.900	34.8%	94.1%	5.9%	0.289	
4.300	43.5%	94.1%	5.9%	0.376	
4.720	43.5%	90.2%	9.8%	0.337	
5.510	52.2%	90.2%	9.8%	0.424	
5.850	52.2%	86.3%	13.7%	0.384	
6.000	56.5%	86.3%	13.7%	0.428	
6.060	56.5%	84.3%	15.7%	0.408	
6.190	60.9%	82.4%	17.6%	0.432	
6.430	78.3%	82.4%	17.6%	0.606	
6.590	87.0%	82.4%	17.6%	0.693	
7.000	100.0%	82.4%	17.6%	0.824	Selected
8.480	100.0%	70.6%	29.4%	0.706	
8.540	100.0%	66.7%	33.3%	0.667	

10.100	100.0%	47.1%	52.9%	0.471	
10.300	100.0%	43.1%	56.9%	0.431	
11.300	100.0%	31.4%	68.6%	0.314	
11.800	100.0%	27.5%	72.5%	0.275	
23.200	100.0%	0.0%	100.0%	0.000	

Table S4(3). Full ROC coordinates—Q3.

GH cut-off (ng/mL)	Sensitivity	Specificity	1 - Specificity	Youden index	Status
1.050	3.7%	100.0%	0.0%	0.037	
2.200	14.8%	100.0%	0.0%	0.148	
2.600	22.2%	100.0%	0.0%	0.222	
2.700	25.9%	100.0%	0.0%	0.259	
3.600	25.9%	97.8%	2.2%	0.238	
4.230	48.1%	97.8%	2.2%	0.460	
4.500	51.9%	97.8%	2.2%	0.497	
4.900	51.9%	93.5%	6.5%	0.453	
5.300	55.6%	93.5%	6.5%	0.490	
5.310	55.6%	91.3%	8.7%	0.469	
6.300	74.1%	91.3%	8.7%	0.654	
6.430	74.1%	89.1%	10.9%	0.632	
6.950	81.5%	89.1%	10.9%	0.706	
7.000	88.9%	89.1%	10.9%	0.780	
7.180	88.9%	87.0%	13.0%	0.758	
7.300	92.6%	87.0%	13.0%	0.795	
7.460	92.6%	84.8%	15.2%	0.774	
7.600	96.3%	84.8%	15.2%	0.811	Selected
8.130	96.3%	82.6%	17.4%	0.789	
8.200	96.3%	78.3%	21.7%	0.746	
11.600	96.3%	39.1%	60.9%	0.354	
11.700	100.0%	37.0%	63.0%	0.370	
13.600	100.0%	21.7%	78.3%	0.217	
14.600	100.0%	17.4%	82.6%	0.174	
22.500	100.0%	0.0%	100.0%	0.000	

Table S4(4). Full ROC coordinates—Q4.

GH cut-off (ng/mL)	Sensitivity	Specificity	1 - Specificity	Youden index	Status
1.280	2.1%	100.0%	0.0%	0.021	
1.600	4.3%	100.0%	0.0%	0.043	
2.300	17.0%	100.0%	0.0%	0.170	
2.400	21.3%	100.0%	0.0%	0.213	
2.500	25.5%	100.0%	0.0%	0.255	
2.570	27.7%	100.0%	0.0%	0.277	
2.800	31.9%	100.0%	0.0%	0.319	

3.290	44.7%	100.0%	0.0%	0.447	
3.400	48.9%	96.4%	3.6%	0.454	
4.540	66.0%	96.4%	3.6%	0.624	
4.600	70.2%	96.4%	3.6%	0.666	
4.900	74.5%	96.4%	3.6%	0.709	
5.040	74.5%	89.3%	10.7%	0.638	
5.070	76.6%	89.3%	10.7%	0.659	
5.100	83.0%	89.3%	10.7%	0.723	
5.650	87.2%	89.3%	10.7%	0.765	
5.700	91.5%	89.3%	10.7%	0.808	
5.990	93.6%	89.3%	10.7%	0.829	Selected
6.000	93.6%	85.7%	14.3%	0.793	
6.450	95.7%	85.7%	14.3%	0.815	
7.040	95.7%	82.1%	17.9%	0.779	
7.510	100.0%	82.1%	17.9%	0.821	
11.600	100.0%	50.0%	50.0%	0.500	
12.900	100.0%	42.9%	57.1%	0.429	
13.500	100.0%	32.1%	67.9%	0.321	
13.900	100.0%	25.0%	75.0%	0.250	
27.100	100.0%	0.0%	100.0%	0.000	

Interpretive note

The sample-derived optimal thresholds were 7.80 ng/mL in Q1, 7.00 ng/mL in Q2, 7.60 ng/mL in Q3, and 5.99 ng/mL in Q4. Thus, the first three quartiles yielded thresholds in a relatively narrow range, whereas the highest BMI SDS quartile yielded a lower threshold of approximately 6 ng/mL. Because GHD diagnosis incorporates GH stimulation testing and all thresholds were derived and evaluated in the same cohort, these results should be regarded as exploratory and hypothesis-generating rather than as validated diagnostic cut-offs.
